# A new species of *Cyrtodactylus* (Squamata, Gekkonidae) from Cambodia’s Prey Lang Wildlife Sanctuary

**DOI:** 10.3897/zookeys.926.48671

**Published:** 2020-04-13

**Authors:** Thy Neang, Adam Henson, Bryan L. Stuart

**Affiliations:** 1 Wild Earth Allies, 77a Street Beton, Bayap Village, Sangkat Phnom Penh Thmei, Khan Sen Sok, Phnom Penh, Cambodia Wild Earth Allies Phnom Penh Cambodia; 2 Wild Earth Allies, 2 Wisconsin Circle, Suite 900, Chevy Chase, Maryland 20815, USA Wild Earth Allies Chevy Chase United States of America; 3 North Carolina Museum of Natural Sciences, 11 West Jones Street, Raleigh, North Carolina 27601, USA North Carolina Museum of Natural Sciences Raleigh United States of America

**Keywords:** *Cyrtodactylusirregularis*, *C.ziegleri*, Mekong River, Phnom Chi, *Sphenomorphuspreylangensis*

## Abstract

*Cyrtodactylusphnomchiensis***sp. nov.** is described from Phnom Chi, an isolated mountain in Prey Lang Wildlife Sanctuary, Kampong Thom Province, Cambodia. The new species is recognized by having a unique combination of morphological characters, including snout-vent length 76.1–80.7 mm; paravertebral tubercles 31–36; ventral scales 45–54; enlarged femoral scales 0–8, without pores; enlarged precloacal scales 7–10, bearing pores 4–5 in males, pits 1–7 in females; the posterior border of nuchal loop unbroken and pointed, bordered anteriorly and posteriorly by a broad yellow or yellowish white band; and yellow spots on top of head. The new species also represents a divergent mitochondrial DNA lineage within the *C.irregularis* complex that is closely related to *C.ziegleri*, but the phylogenetic relationships among the new species and two divergent mitochondrial subclades within *C.ziegleri* are not resolved based on available sequence data. *Cyrtodactylusphnomchiensis***sp. nov.** is the only member of the *C.irregularis* complex known to occur west of the Mekong River. The new species may be endemic to Phnom Chi, and likely faces imminent conservation threats.

## Introduction

Bent-toed Geckos of the genus *Cyrtodactylus* Gray are one of the most species-diverse genera of gekkonid lizards, with 292 recognized species (Uetz et al. 2020). Much of the diversity within *Cyrtodactylus* has been described only during the past decade and from mainland Southeast Asia (Brennan et al. 2017; Uetz et al. 2020), and many of these newly-recognized species are thought to be highly localized with extremely narrow geographic ranges (e.g., Nazarov et al. 2012; Luu et al. 2016; Grismer et al. 2017; Murdoch et al. 2019).

*Cyrtodactylusirregularis* (Smith, 1921) was originally described from the Langbian Plateau near Da Lat, southern Vietnam. For nearly a century, *C.irregularis* was treated as a single, geographically widespread, but morphologically variable species. Recent taxonomic studies on variation in morphology and, usually, the mitochondrial cytochrome *c* oxidase subunit I (COI) gene (Brennan et al. 2017) have revealed that *C.irregularis* actually represents a complex of at least 19 species distributed in southern and central Vietnam, eastern Cambodia, and southern Laos (Nguyen et al. 2013, 2017; Pauwels et al. 2018). These include 18 named species from Vietnam recognized by Pauwels et al. (2018), as well as *C.buchardi* David, Teynie & Ohler, 2004 from southern Laos, a species that has been hypothesized to be a member of this complex (Ngo and Chan 2010; Nguyen et al. 2013, 2017) but that remains phylogenetically untested owing to lack of molecular data. The monophyly of the *C.irregularis* group has been demonstrated by phylogenetic analysis of the COI gene from most of the species in the complex (Nazarov et al. 2012; Nguyen et al. 2013, 2014, 2017; Luu et al. 2017; Schneider et al. 2014).

During field surveys by Wild Earth Allies in June–July 2019, five specimens of the *C.irregularis* complex were collected in Cambodia on the western side of the Mekong River at Phnom Chi (Mountain) in Prey Lang Wildlife Sanctuary, Kampong Thom Province. Herein, we investigate the taxonomic status of the Phnom Chi specimens through comparisons of morphological and mitochondrial DNA data with other members of the *C.irregularis* complex.

## Materials and methods

### Sampling

Field work was conducted both day and night to search microhabitats for amphibians and reptiles at Phnom Chi. Specimens were collected by hand and kept overnight in individual plastic or cloth bags for photographing the following day. Specimens were euthanized by cardiac injection of high concentration of tricaine methanesulfonate (MS-222) and fixed in 10% formalin after preserving liver tissue in 20% DMSO-salt saturated storage buffer. After a minimum of three days of formalin-fixation, the specimens were soaked in water for six hours to remove formalin, and transferred to 70% ethanol for permanent storage. Specimens were deposited in the herpetological collection at the Centre for Biodiversity Conservation, Royal University of Phnom Penh, Cambodia (**CBC**). Comparative data were taken from original species descriptions and the expanded descriptions of *C.irregularis* by Nazarov et al. (2008) and *C.buchardi* by Teyníe and David (2010).

### Morphological analyses

Morphometric and meristic characters were measured and counted using a Nikon SMZ 645 dissecting microscope. Measurements were taken by hand with digital calipers to the nearest 0.1 mm (ratios calculated to 0.001). Measured characters were:

**AG** Axilla-groin distance, measured from the posterior margin of forelimb at its insertion point on the body to the anterior margin of hind limb at its insertion point on the body;

**CrusL** Crus length, measured from the knee to the base of the heel;

**EarDH** Ear diameter in horizontal distance, measured as the horizontal distance between anterior and posterior margins of the ear opening;

**EarDV** Ear diameter in vertical distance, measured as the vertical distance between dorsal and ventral margins of the ear opening;

**END** Eye-nostril distance, measured from the anterior margin of eye to the posterior margin of nostril;

**ESD** Eye-snout distance, measured from the anterior margin of eye to the tip of snout;

**EyeD** Eye diameter, measured as the horizontal distance from the anterior to the posterior margins of the eyeball;

**Eye-EarD** Eye-ear distance, measured from the posterior margin of eye to the anterior margin of ear opening;

**ForeL** Forearm length, measured from the posterior margin of elbow while flexed 90° to the wrist inflection;

**HeadD** Head depth, measured as the maximum depth of head from the occiput to the throat;

**HeadL** Head length, measured from the tip of snout to the posterior margin of the retroarticular process of the lower jaw;

**HeadW** Head width, measured as the maximum head width at the corners of the jaws;

**IOD** Interorbital distance, measured as the shortest distance between the anterior corners of the eyes;

**IND** Internarial distance, measured as the shortest distance between the nostrils;

**SVL** Snout to vent length, measured from the tip of the snout to the vent;

**TaL** Tail length, measured from the vent to the tip of the tail;

**TaW** Tail width, measured at the base of the tail immediately posterior to the post-cloacal swelling.

Scale counts are reported in right and left (R, L) order. The presence, absence and/or numbers of the following characters were recorded:

**EFS** Enlarged femoral scales;

**EPrecS** Enlarged precloacal scales;

**FP** Femoral pores;

**InL** Infralabials, counted as the number of scales from the first lower labial scale immediately posterior to mental to the last scale below posterior edge of the eyeball;

**LDRT** Longitudinal dorsal rows of enlarged tubercles, counted as the number of tubercles transversely across the dorsum between ventrolateral folds;

**PrecG** Precloacal groove;

**PrecP** Precloacal pores;

**PostPSR** Post precloacal scale rows;

**PostSP** Post cloacal spur;

**PVT** Paravertebral tubercles, counted as the number of enlarged tubercles in a straight line between limb insertions left of the vertebral column;

**SDLF4** Subdigital lamellae beneath fourth finger, counted as the number of both expanded proximal subdigital lamellae from the base to the largest scale on the digital inflection, and unmodified distal lamellae beneath fourth finger to the claw sheath;

**SDLT4** Subdigital lamellae on fourth toe, counted as the number of expanded proximal subdigital lamellae from the base to the largest scale on digital inflection and unmodified distal subdigital lamellae beneath fourth toe to the claw sheath;

**SL** Supralabials, counted as the number of scales from the first upper labial scale immediately posterior to rostral to the last scale below posterior edge of the eyeball;

**VS** Ventral scales, counted as the number of scales transversely across the ventral surface at midbody between ventrolateral folds.

### Molecular analyses

Total genomic DNA was extracted from preserved liver tissue of two Phnom Chi specimens (CBC 03003–04) using the DNeasy Blood and Tissue Kit (Qiagen). A 658 bp fragment of mitochondrial (mt) DNA that encodes part of the COI gene was amplified in a 25 ul reaction by the polymerase chain reaction (PCR; 35 cycles of 95° 30s, 53 °C 40s, 72° 90s) and sequenced using the primers VF1d and VR1d (Ivanova et al. 2006). PCR products were cleaned using ExoSAP-IT (Applied Biosystems) and sequenced in both directions by direct double strand cycle sequencing using the BigDye Terminator version 3.1 Cycle Sequencing Kit on a 3130 DNA Analyzer (Applied Biosystems). Sequences were edited with Sequencher version 5.4.6 (Gene Codes) and deposited in GenBank under accession numbers MT066405–MT066406.

All available *Cyrtodactylus*COI sequences (*n* = 453), and the outgroup *Hemidactylusfrenatus* (GenBank accession GQ245970), were downloaded from GenBank on 1 October 2019. The downloaded sequences were aligned and visually inspected in Sequencher to ensure that insertion-deletions did not disrupt the coding region. Preliminary phylogenetic analysis (not shown) was performed on the alignment under the parsimony criterion using a heuristic search with equal weighting of nucleotide substitutions in PAUP* version 4.0a165 (Swofford 2003). Those *Cyrtodactylus* sequences that clustered in the clades around the Phnom Chi samples (= *C.irregularis* group) in a strict consensus of the equally most parsimonious trees were retained in the alignment. Exemplar sequences of other major clades were also retained to represent known phylogenetic diversity within *Cyrtodactylus*, including *C.auribalteatus* (GenBank accession AP018116), *C.badenensis* (KF929505), *C.chanhomeae* (MF169908), *C.condorensis* (MF169910), *C.interdigitalis* (MF169919), *C.intermedius* (MF169920), *C.jellesmae* (MF169923), *C.peguensis* (AP018114), *C.russelli* (MF169938), and *C.thirakhupti* (AP018115).

The resulting pruned COI alignment contained 270 taxa and 717 characters, with no insertion-deletions. The alignment was partitioned by codon position, and the best-fit partitioning scheme and models of sequence evolution were selected using PartitionFinder 2 (Lanfear et al. 2017). Two partitions were selected, with the first and second codon positions merged into a single partition under the model TVM+I+G, and the third codon position under the model GTR+G. Four independent partitioned Bayesian analyses were performed using MrBayes 3.2.7a (Ronquist et al. 2012) on the Cyberinfrastructure for Phylogenetic Research (CIPRES) Science Gateway version 3.3 (Miller et al. 2010). In each analysis, four chains were run for 20 million generations using the default priors, the chain temperature was set to 0.1, trees were sampled every 4,000 generations, and the first 25% of trees were discarded as ‘burn-in’. The resulting trace plots were viewed using Tracer v.1.7 (Rambaut et al. 2018). A 50% majority-rule consensus of the post burn-in trees was constructed to calculate the posterior probabilities of nodes. Nodes with posterior probabilities ≥ 0.95 were considered to be statistically supported. Uncorrected pairwise distances were calculated using PAUP* version 4.0a165 (Swofford 2003).

## Results

### Morphological analyses

The Phnom Chi specimens could not be referred to any other named members of the *C.irregularis* complex owing to having a unique combination of morphological characters. These characters included body size such as having a relatively long body and tibia; scalation, such as the number of subdigital lamellae under the fourth finger and fourth toe, number of longitudinal dorsal and paravertebral rows of tubercles, number of ventral scales, number of enlarged precloacal scales and associated pores (in males) and pits (in females), absence of pores in their enlarged femoral scales, and size of the median subcaudal scale rows from other species in the complex; and pattern and coloration, including an unbroken nuchal loop bordered anteriorly and posteriorly by a broad yellow or yellowish white band, three or four dark brown body bands, and two or three yellowish white or light brown body bands, about half the width of the brown body bands, and yellow spots on top of the head.

### Molecular analyses

The standard deviation of split frequencies among the four Bayesian runs was 0.006260 and the Estimated Sample Sizes (ESS) of parameters were ≥ 1,606, indicating that the four runs were sufficiently sampled and had converged. The Phnom Chi specimens represented a distinct mitochondrial lineage that did not match any other named species (Fig. 2). The Phnom Chi lineage was recovered with strong support (Bayesian posterior probability 1.00) to be phylogenetically nested within a clade containing two mitochondrial subclades of *C.ziegleri* (subclades Z1 and Z2; Fig. 2), but the relationships among the Phnom Chi lineage and the two subclades of *C.ziegleri* were unresolved, rendering *C.ziegleri* non-monophyletic (Fig. 2). The clade containing the Phnom Chi lineage and the two subclades of *C.ziegleri* was recovered with strong support (Bayesian posterior probability 1.00) to be sister to *C.bugiamapensis* (Fig. 2).

The Phnom Chi samples had uncorrected *p*-distances in COI of 4.3–6.2% from *C.ziegleri* (all samples) and 7.0–8.6% from *C.bugiamapensis*. *Cyrtodactylusziegleri* (all samples) had uncorrected *p*-distances of 6.7–8.5% from *C.bugiamapensis*. *Cyrtodactylusziegleri* subclade Z1 had uncorrected *p*-distances of 4.7–5.2% from *C.ziegleri* subclade Z2.

**Figure 1.**
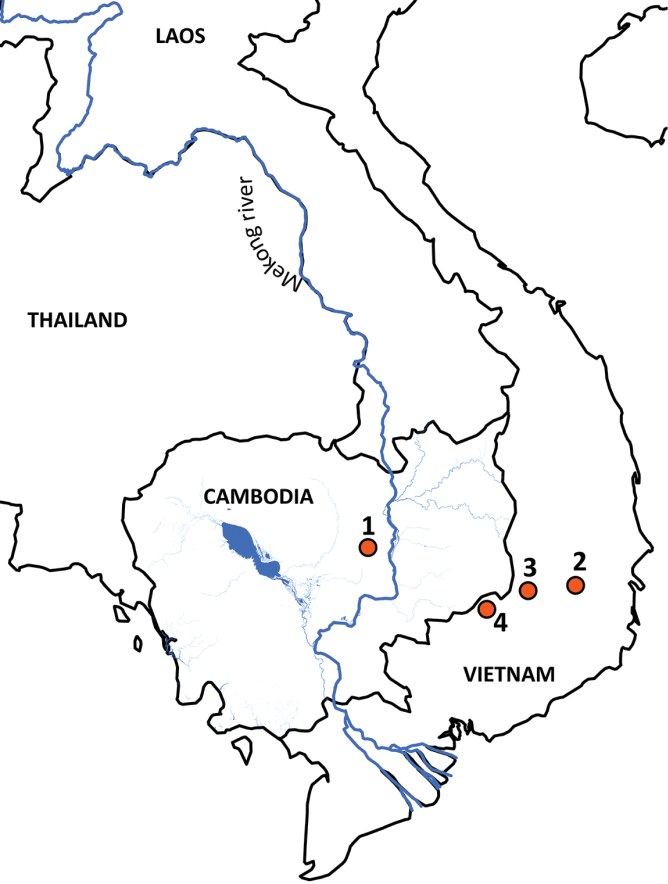
Map illustrating (1) the type locality of *Cyrtodactylusphnomchiensis* sp. nov. at Prey Lang Wildlife Sanctuary, Kampong Thom Province, Cambodia; (2) the type locality of *C.ziegleri* at Chu Yang Sin National Park, Dak Lak Province, Vietnam (Nazarov et al. 2008); (3) the second known locality of *C.ziegleri* at Nam Nung Nature Reserve, Dak Nong Province, Vietnam (Nguyen et al. 2013); and (4) the type locality of *C.bugiamapensis* at Bu Gia Map National Park, Binh Phuoc Province, Vietnam (Nazarov et al. 2012).

**Figure 2.**
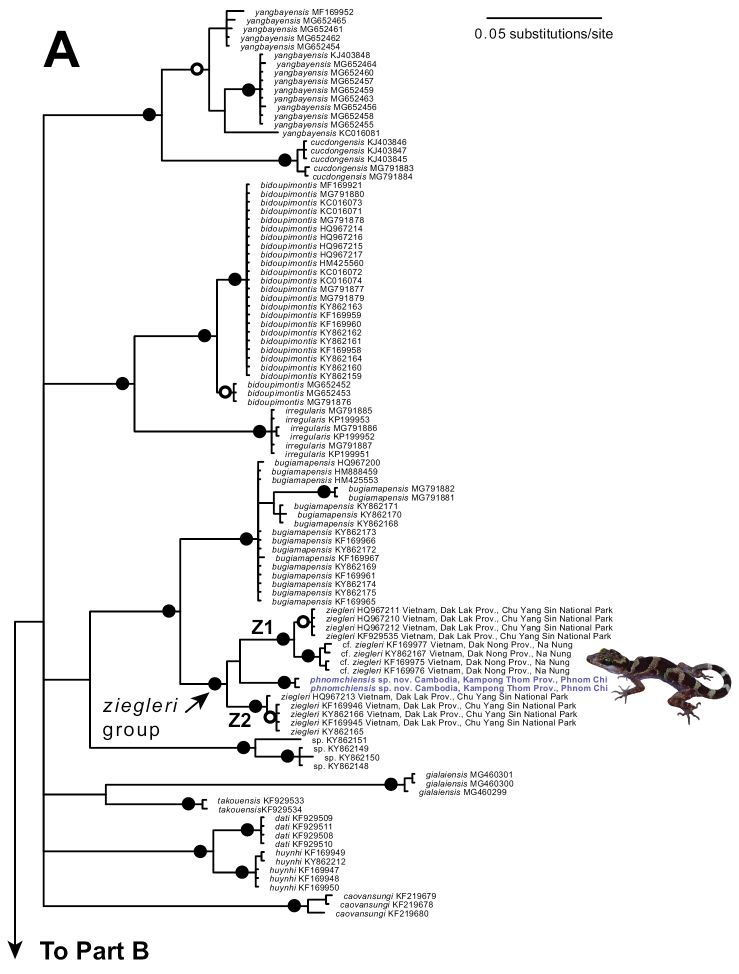
Upper (**A**) and lower (**B**) portions of a fifty percent majority-rule consensus phylogram resulting from partitioned Bayesian analysis of 717 aligned characters of the mitochondrial cytochrome *c* oxidase subunit I (COI) gene from geckos in the *Cyrtodactylusirregularis* group. The outgroup *Hemidactylusfrenatus* (GenBank accession GQ245970) and exemplars of other *Cyrtodactylus* clades including *C.auribalteatus* (GenBank accession AP018116), *C.badenensis* (KF929505), *C.chanhomeae* (MF169908), *C.interdigitalis* (MF169919), *C.intermedius* (MF169920), *C.jellesmae* (MF169923), *C.peguensis* (AP018114), *C.russelli* (MF169938), and *C.thirakhupti* (AP018115) were also included in the analysis (not shown). Black circles at nodes indicate Bayesian posterior probabilities ≥ 0.99, and open circles at nodes indicate Bayesian posterior probabilities ≥ 0.95. Numbers at terminal tips are GenBank accession numbers.

**Figure 2.**
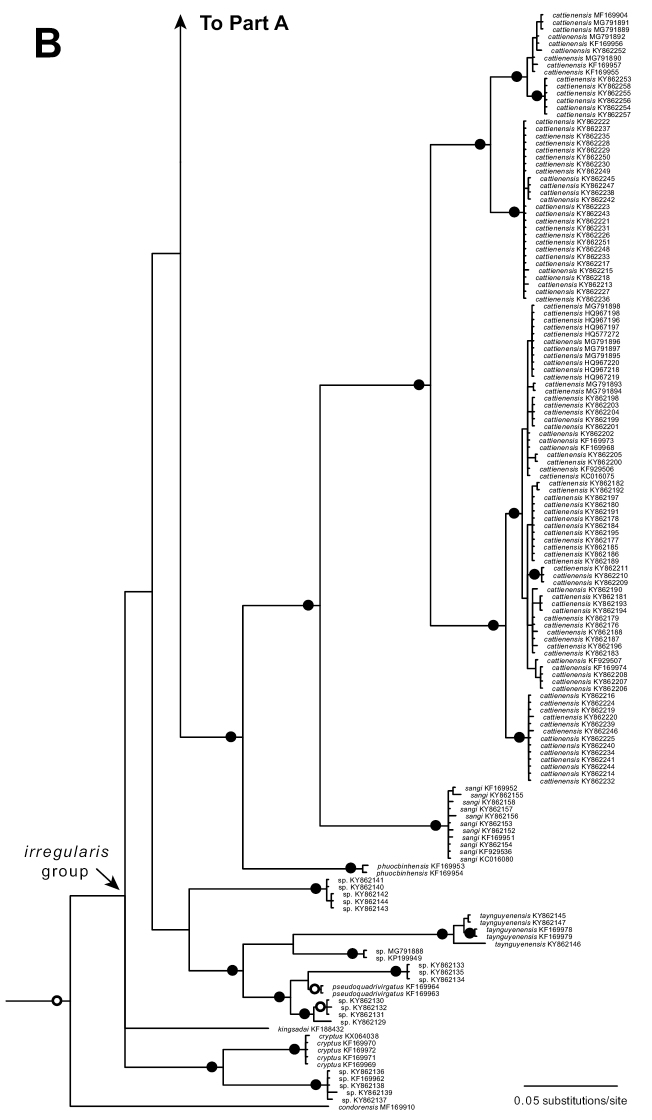
Continued.

### Species description

On the basis of their distinctiveness in morphology and mitochondrial DNA, including from *C.ziegleri* to which they are phylogenetically related (but exact relationship unresolved; Fig. 2), and further corroborated by their geographic distance to any other named members in the complex (and the only member known from west of the Mekong River), the Phnom Chi specimens are hypothesized to represent a distinct species, described herein as:

#### Cyrtodactylus phnomchiensis sp. nov.

Taxon classificationAnimaliaSquamataGekkonidae

2E84EE8A-C166-5CDA-B3C1-E18270D1D3E0

http://zoobank.org/103B12F4-6D6F-4928-85A5-A927A81225FE

**Figure 3.**
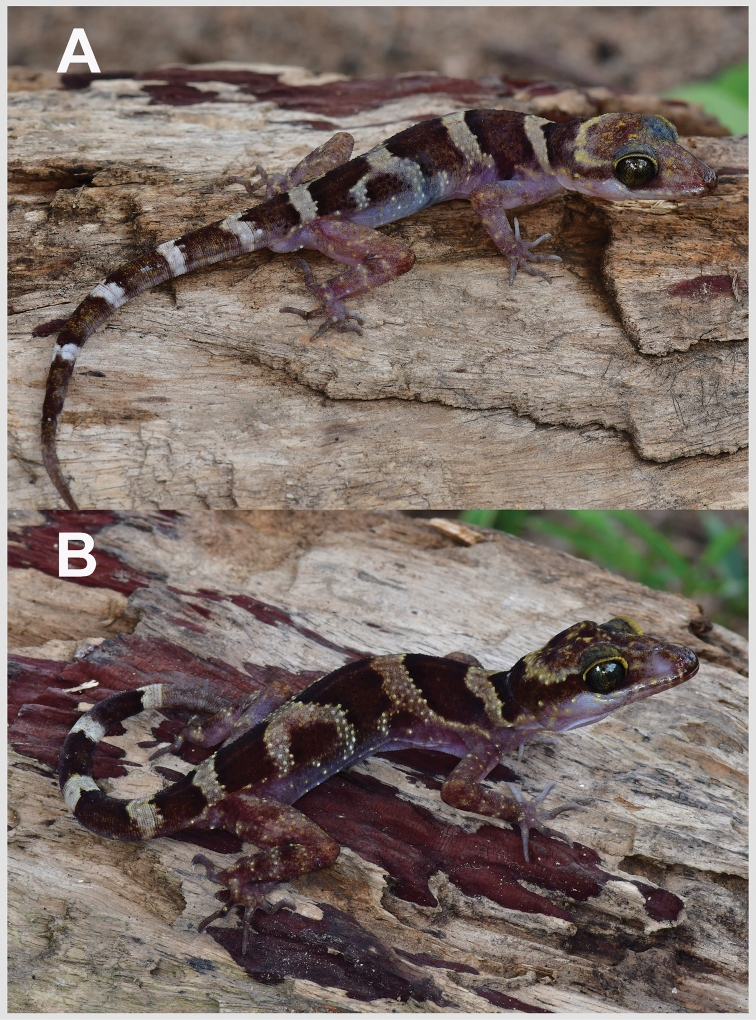
*Cyrtodactylusphnomchiensis* sp. nov. in life. **A** Male holotype CBC 03012 and **B** female paratype CBC 03013.

**Figure 4.**
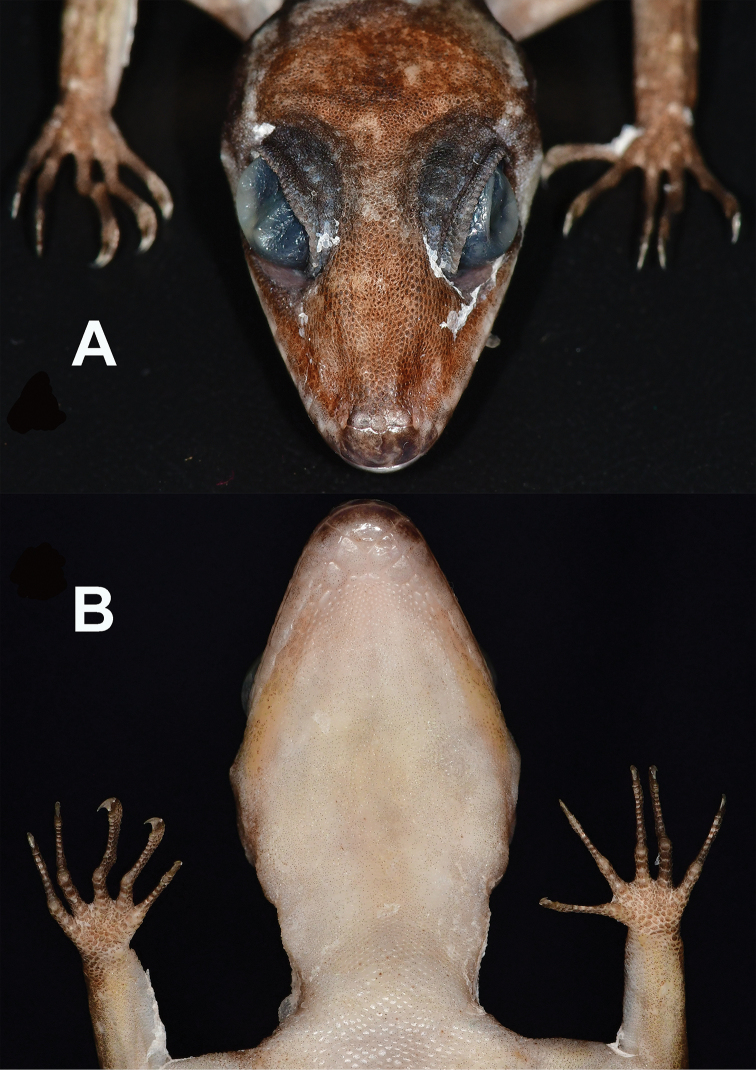
Head of male holotype CBC 03012 of *Cyrtodactylusphnomchiensis* sp. nov. in preservative. **A** Dorsal view illustrating the rostral, supranasal and internsupranasal scales **B** ventral view illustrating the mental and postmental scales.

**Figure 5.**
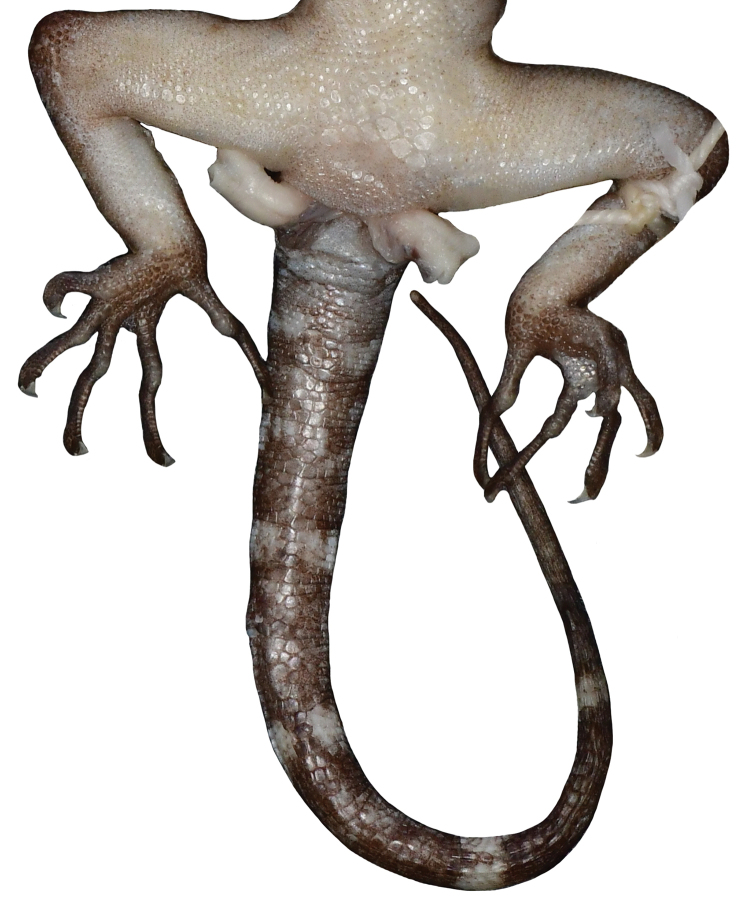
Cloacal region of male holotype CBC 03012 of *Cyrtodactylusphnomchiensis* sp. nov. in preservative illustrating the enlarged precloacal scales, enlarged femoral scales, and not enlarged subcaudal scales.

**Figure 6.**
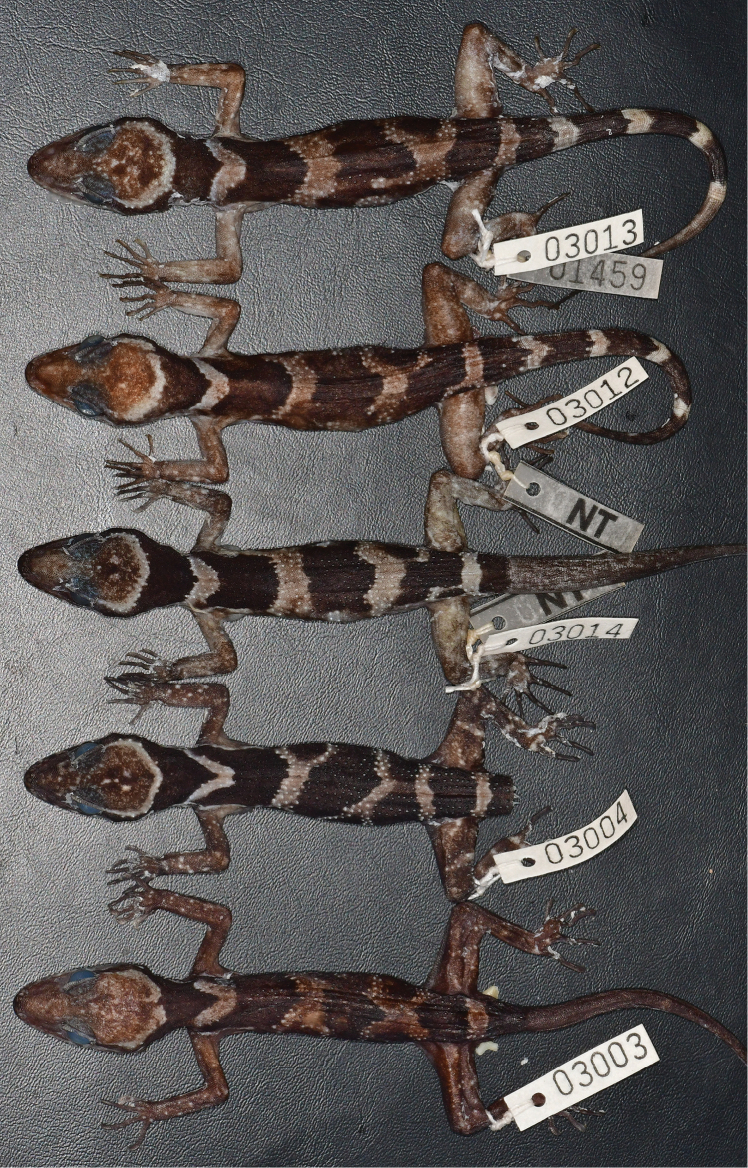
Dorsal view of the type series of *Cyrtodactylusphnomchiensis* sp. nov. in preservative.

Figures 3 , 4 , 5 , 6

##### Holotype.

CBC 03012, adult male (Fig. 3), Cambodia, Kampong Thom Province, Sandan District, Phnom Chi, Prey Lang Wildlife Sanctuary, 12°56'11.6"N, 105°39'17.1"E, 237 m elevation, collected on 18 July 2019 by Thy Neang and En E.

##### Paratypes.

All from Cambodia, Kampong Thom Province, Sandan District, Phnom Chi, Prey Lang Wildlife Sanctuary: CBC 03003, adult male, 12°56'09.2"N, 105°39'12.7"E, 269 m elevation, coll. 13 June 2019 by Thy Neang; CBC 03004, adult female, 12°56'09.7"N, 105°39'14.4"E, 271 m elevation, coll. 13 June 2019 by Thy Neang; CBC 03013, adult female, same data as holotype; CBC 03014, adult female, same data as holotype except 12°56'08.7"N, 105°39'12.6"E, 284 m elevation.

##### Etymology.

The specific epithet is taken from the type locality of Phnom Chi and the Latin suffix -*ensis* meaning “originating from.” The specific epithet is masculine in agreement with the gender of *Cyrtodactylus*.

##### Diagnosis.

*Cyrtodactylusphnomchiensis* sp. nov. is distinguished from the 19 other named species in the *C.irregularis* group (Ngo and Chan 2010; Nguyen et al. 2013, 2017; Pauwels et al. 2018) by having the combination of SVL 76.1–80.7 mm; relatively long body, AG/SVL 0.451–0.481; relatively long tibia, CrusL/SVL 0.172–0.200; subdigital lamellae on fourth finger 18–20; subdigital lamellae on fourth toe 20–23; longitudinal dorsal rows of tubercles 18–20; paravertebral rows of tubercles 31–36; ventral scales 45–54; enlarged femoral scales 0–8, without pores; enlarged precloacal scales 7–10, bearing pores 4 or 5 in males, pits 1–7 in females; precloacal groove absent; median row of transverse subcaudal scales only slightly enlarged; posterior border of nuchal loop unbroken and pointed, bordered anteriorly and posteriorly by broad yellow or yellowish white band; dark brown body bands 3 or 4, the first intact, the second, third and fourth more irregular, alternating with two or three yellowish white or light brown body bands, about half the width of dark brown body bands; and yellow spots on top of head.

##### Description of holotype.

Adult male with SVL 76.1 mm; head slightly elongate, HeadL 22.1 mm, about 30% of SVL, moderately widened, HeadW 14.1 mm, HeadW/HeadL 0.64, slightly depressed, HeadD 9.4 mm, HeadD/HeadL 0.43, distinct from neck, triangular in dorsal profile; snout rather elongated, rounded in rostral region, ESD 9.0 mm, slightly less than HeadD, ESD/HeadL 0.41, frontonasal region flattened, prefrontal region slightly concave, forming elongated medial rostral groove, canthus rostralis flattened, slightly angled between loreal region and rostral groove; lores posterior to nostrals depressed, anterior to orbit flattened; eye large, eyeball rounded, slightly protruding, EyeD 5.1 mm, shorter than the distance between eye and ear, Eye-EarD 5.7 mm, pupil vertical, covered by crenellate supraciliaries; ear opening oval, deeply sunk, rather small, elongated in oblique position, EarDV 1.2 mm, almost twice longer than its diameter in horizontal position, EarDH 0.7 mm; rostral large, subrectangular, height 1.9 mm, shorter than its width 3.6 mm, medially divided dorsally by a suture, reaching to about half way of rostral height, in contact with 1^st^SL and nostrils laterally, supranasals and internasal dorsally (Fig. 4); nostrils pieced at anterior angle of snout, directed lateroposteriorly, surrounded by rostral anteriorly, 1^st^SL ventrally, supranasals dorsally, and three small postnasal scales; internarial distance narrow, IND 2.9 mm; supranasals subrectangular, separated by intersupranasal, slightly smaller in size, in contact with rostral anteriorly, nostrals laterally, four small scales posteriorly; intersupranasal single, subpentagonal, slightly protruding rostral, in contact with two small scales posteriorly; interorbital rather narrow, IOD 5.5 mm, longer than EyeD, slightly shorter than Eye-EarD 5.7 mm; supralabials (12R, 13L), subrectangular anteriorly, circular shape posteriorly, anterior SL separated from small scales on loreal region by row of slightly enlarged scales; infralabials (9R, 9L), larger than SL, first InL bordered by mental anteriorly, first postmental ventrally, second InL bordered by second enlarged postmental, enlarged chin shield scale ventrally, 3–7^th^InL bordered by a row of slightly enlarged chin shield scales ventrally; mental large, triangular, width 3.3 mm in width, 2.3 mm in length, in contact with first InL laterally, two pairs postmentals posteriorly; the first pair largest, subrectangular, in broad contact medially, second pair enlarged, half the size of the first pair, separated by four smaller gular scales medially, in contact with smaller scales posteriorly (Fig. 4). Scales on frontonasal, prefrontal, loreal regions small, almost homogenous, slightly larger than those on top of head; scales on occiput intermixed with scattered larger, more rounded, conical tubercles, more prominent tubercles on region between orbit and area above ear opening, a noticeably larger tubercle (compared to those surrounding) at the corner of jaw.

Body slightly slender, AG 36.6 mm, nearly half SVL, AG/SVL 0.481 with well-defined narrow vertebral furrow posteriorly; scales on dorsum small, mostly homogenous, granular, interspersed with larger, low, weakly keeled, irregularly arranged, tubercles; longitudinal dorsal rows of enlarged tubercles approximately 18; paravertebral tubercles 32; tubercles on nape within dark brown nuchal loop, anterior dorsal surface at level above shoulder smaller, more rounded, sparser than those on mid-dorsum and posterior dorsal surface, more prominent, being denser, weekly keeled, more regularly arranged on sacral and tail base region; tubercles on lateral body sparsely; ventral scales small, not imbricate, those near midline larger than lateral and dorsal scales; scales on throat and gular region the smallest; faint ventrolateral folds with few emerged tubercles; ventral scales at midbody between ventrolateral folds 47; precloacal region moderately enlarged, a few rows of enlarged precloacal non-pore bearing scales anterior to pore bearing precloacal scales; enlarged precloacal scales 7, in angular series, bearing 5 pores, terminal scale on each side poreless; post precloacal scale rows 3, the first row immediately posterior to enlarged precloacal pore-bearing scales with six scales in angular series, the second row with four scales in angular series, the third row with three scale in straight line, the medial scale largest; femoral scales slightly enlarged (8R, 8L), distal scales more than twice the size of proximal scales, all smaller than those of pore-bearing precloacal scales, separated from precloacal scales by diastema; precloacal groove absent; fully everted hemipenes thick, 5.9 mm in length, two penes at each sheath, two sockets posterior to hemipenal bases (Fig. 5).

Limbs rather slender; digits with strongly inflected interphalangeal joints; forelimbs bearing five relatively slender fingers, moderately bowed, ending with curved claws, ForeL/SVL 0.162; expanded proximal subdigital lamellae on fourth finger 6, unmodified distal subdigital lamellae on fourth finger 12, total subdigital lamellae on fourth finger 18; hind limbs bearing five relatively slender toes, strongly bent, ending with curved claws, CrusL/SVL 0.176; expanded proximal subdigital lamellae on fourth toe 7, unmodified distal subdigital lamellae on fourth toe 15, total subdigital lamellae on fourth toe 22; all digits lacking scansorial setae on ventral surface; scales on limbs small, interspersed with larger, low, conical, weakly keeled tubercles; scales on palmar and plantar surfaces small.

Tail moderately wide anteriorly, TaW 5.5 mm, segmented, cylindrical, becoming slender toward tip, regenerated posteriorly; dorsal caudal longitudinal tubercle rows at base of tail 8; 2 transverse rows of dorsal caudal tubercles at posterior margin of third band on tail, 22.7 mm from tail base; vertebral caudal surface with scattered bump at approximate intervals of 3 mm; subcaudal scale rows smooth, small, differing in size and irregular in shape, usually alternating between a single slightly enlarged and two smaller scales, 2 or 3 times larger than neighboring lateral caudal scales (Fig. 5).

##### Color of holotype in life

(Fig. 3). Dorsal surface, nape, and tail yellowish white to light brown; top of head with yellowish spots; interorbital region, rostral and loreal regions lighter brown with scattered yellowish scales; eye ring yellowish; rostral, mental lighter brown; supralabials, corner of jaw, and region extending through dorsal margin of ear opening to shoulder yellowish; nuchal loop with large dark brown band, pointed, extending between posterior margins of eyes, bordered anteriorly by broad yellow band along upper edge of dark brown nuchal loop, posteriorly by yellowish white band; three dorsal dark brown bands on body, the first more regular, the second and third bands irregular, interrupted by white irregular blotches; all dark brown bands ending near to mid-flank region, bordered below by lighter brown extending to lateral folds; dark brown body bands bordered by yellowish white or light brown bands about half the width of dark brown body bands, the last light brown band ending on tail base; anterior and posterior margins of body bands with darker brown coloration; dark brown bands 6 on regenerated tail, margins at tail base darker; white bands on tail 5, nearly encircling the tail except subcaudal scale row; subcaudal scales lighter brown than dorsal caudal scales; limbs lighter brown with orangish or yellowish on enlarged tubercles; ventral surfaces between ventrolateral folds, chin, throat, and limbs white with tiny black dots on tip of scales; ventral surfaces of fingers and toes dark brown. In preservative, all yellowish, yellowish white or orange coloration faded to white, cream, or light brown (Fig. 6).

##### Variations.

Morphometric and meristic characters of the type series are presented in Table 1. The paratypes generally resemble the holotype (Fig. 6), except as follows. CBC 03003 has four enlarged post precloacal scale rows in an angular series, the last row with only a single enlarged scale. CBC 03004 and CBC 03014 have darker brown body bands. CBC 03003–03004 have a more pointed nuchal loop. CBC 03003 has more dense dark dots causing ventral surfaces to be darker brown. CBC 03003 has more slender, fully everted hemipenes. Females have enlarged precloacal scales with pits rather than pores.

**Table 1.** Mensural, meristic and color pattern characters of *Cyrtodactylusphnomchiensis* sp. nov. Abbreviations defined in the text. All specimens have regenerated portions of tails (*).

Voucher specimen	CBC 03012	CBC 03003	CBC 03004	CBC 03013	CBC 03014	Range
Type status	Holotype	Paratype	Paratype	Paratype	Paratype	
Sex	Male	Male	Female	Female	Female	
SVL	76.1	79.0	77.3	80.7	76.7	76.1–80.7
TaL*	75.1	56.9	66.6	79.1	64.6	56.9–79.1
TaW	5.5	4.4	5.3	5.6	5.7	4.4–5.7
TaW/SVL	0.072	0.056	0.069	0.069	0.074	0.056–0.74
ForeL	12.3	13.2	12.1	13.7	11.7	11.7–13.7
ForeL/SVL	0.162	0.167	0.157	0.170	0.153	0.153–0.170
CrusL	13.4	14.9	13.8	16.1	13.2	14.2–16.6
Crus/SVL	0.176	0.189	0.179	0.200	0.172	0.172–0.200
AG	36.6	36.1	35.3	36.4	35.4	35.3–36.6
AG/SVL	0.481	0.457	0.457	0.451	0.462	0.451–0.481
HeadL	22.1	23.5	22.2	23.6	23.4	22.1–23.6
HeadL/SVL	0.290	0.297	0.287	0.292	0.305	0.287–0.305
HeadW	14.1	14.5	13.7	15.2	13.7	13.7–15.2
HeadD	9.4	9.2	8.6	9.8	8.6	8.6–9.8
EyeD (eye diameter)	5.1	5.1	4.8	4.8	4.5	4.5–5.1
EyeD/SVL	0.067	0.065	0.062	0.059	0.059	0.059–0.067
Ear-EyeD (eye-ear distance)	5.7	6.1	6.0	6.5	5.7	5.7–6.5
ESD (eye-snout distance)	9.0	9.5	9.0	9.9	9.3	9.0–9.9
ESD/SVL	0.118	0.120	0.116	0.123	0.121	0.116–0.123
END (eye-nostrial distance)	6.6	6.9	6.4	7.0	7.0	6.4–7.0
IO (interorbital distance)	5.5	4.8	5.0	5.7	5.2	4.8–5.7
IND (internarial distance)	2.9	2.8	2.6	2.9	2.7	2.6–2.9
EarDV (vertical)	1.2	1.3	1.2	1.3	1.3	1.2–1.3
EarDH (horizontal)	0.7	1.1	0.7	0.7	0.7	0.6–1.1
Intersupranasal scales	1	1	1	1	1	1
Supralabials (SL)	12R/13L	11R/11L	11R/11L	12R/12L	12R/13L	11–13
Infralabials (InL)	9R/9L	10R/9L	10R/10L	10R/9L	10R/10L	8–10
PVT	32	31	36	34	32	31–36
LDRT	18	20	20	20	19	18–20
VS	47	47	52	45	54	45–54
Median subcaudal scales slightly enlarged	yes	yes	yes	yes	yes	yes
SDLF4	18	20	18	19	19	18–20
SDLT4	22	23	20	21	21	20–23
EFS	8R8L	3R/3L	0	7R/6L	0	0–8
FP	0	0	0	0	0	0
EPrecS	7	9	7	10	9	7–10
PrecP	5	4	4	1	7	1–7
PrecG	0	0	0	0	0	0
PostPSR	3	4	3	3	3	3–4
PostSP	4	3	4	4	3	3–4
Number of dark brown body bands	3	3	4	3	3	3–4
Femoral and precloacal scales continuous	no	no	no	no	no	no
Yellowish spots on top of head	yes	yes	yes	yes	yes	yes
Posterior border of nuchal loop pointed	yes	yes	yes	yes	yes	yes
First body band complete	yes	yes	yes	yes	yes	yes
Second to fourth body bands more irregular	yes	yes	yes	yes	yes	yes
Yellowish white or light brown bands about half the width of dark brown body bands	yes	yes	yes	yes	yes	yes
Number of yellowish white or light brown body bands	2	2	3	2	2	2–3
Yellowish spot above ear opening	yes	yes	yes	yes	yes	yes
Enlarged tubercle at corner of jaw	yes	Yes	yes	yes	yes	yes

##### Distribution and natural history.

**Figure 7.**
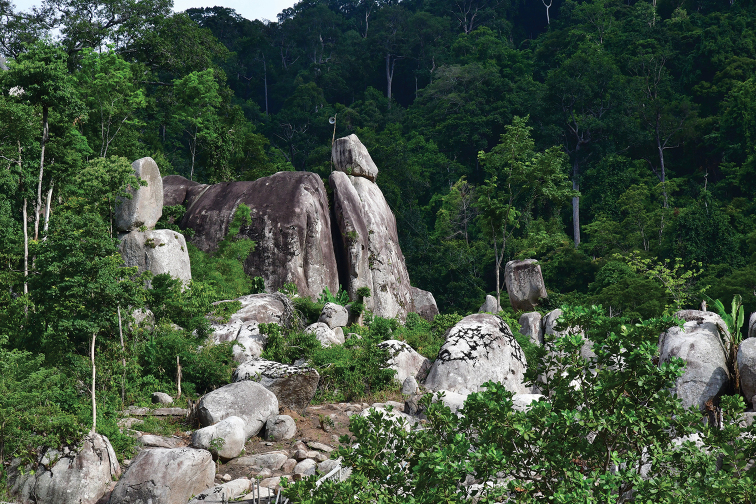
Habitat at Phnom Chi, the type locality of *Cyrtodactylusphnomchiensis* sp. nov.

The new species is known only from the type locality at Phnom Chi in Prey Lang Wildlife Sanctuary, Kampong Thom Province, Sandan District, Cambodia. All individuals were found at night between 2001–2147 hr in evergreen-large dipterocarp dominated forest associated with rocky terrain (Fig. 7). The holotype CBC 03012 was found on a rock face following evening rain, paratypes CBC 03013–14 were on boulders following evening rain, paratype CBC 03003 was on leaf litter along a forest trail, and paratype CBC 03004 was on a rock wall at the entrance to a cave. Only five individuals were found during five-survey nights, suggesting the species is relatively uncommon. None were encountered during a brief survey by NT in the wet season of 2014 (Hayes et al. 2015). The new species is the only member of the C. *irregularis* complex known to occur west of the Mekong River (Nguyen et al. 2017; Pauwels et al. 2018).

##### Comparisons.

*Cyrtodactylusphnomchiensis* sp. nov. is distinguishable from all 19 other members of the *C.irregularis* group by a unique combination of morphological characters (and in mitochondrial DNA; Fig. 2).

*Cyrtodactylusphnomchiensis* sp. nov. differs from *C.bidoupimontis* Nazarov, Poyarkov, Orlov, Phung, Nguyen, Hoang & Ziegler, 2012 by having ventral scales 45–54 (vs. 38–43 in *bidoupimontis*), precloacal pits in females 1–7 (vs. absent in *bidoupimontis*), dark brown body bands larger than yellowish white or light brown dorsal bands (vs. dark brown bands, when present, narrower than light yellow dorsal bands in *bidoupimontis*), and distinct large yellow band on anterior margin of dark brown nuchal loop (vs. narrow light margin in *bidoupimontis*), and yellow spots on top of head (vs. dark spots in *bidoupimontis*).

*Cyrtodactylusphnomchiensis* sp. nov. differs from *C.buchardi* by having SVL 76.1–80.7 mm (vs. 60–65 mm in *buchardi*), SDLF4 18–20 (vs. 14 in *buchardi*), SDLT4 20–23 (vs. 12–14 in *buchardi*), ventral scales 45–54 (vs. 30 in *buchardi*), LDRT 18–20 (vs. 25 in *buchardi*), precloacal pores in males 4–5 (vs. 9 in *buchardi*), and irregular dorsal body bands (vs. blotches in *buchardi*).

*Cyrtodactylusphnomchiensis* sp. nov. differs from *C.bugiamapensis* Nazarov, Poyarkov, Orlov, Phung, Nguyen, Hoang & Ziegler, 2012 by having LDRT 18–20 (vs. 20–24 in *bugiamapensis*), ventral scales 45–54 (vs. 36–46 in *bugiamapensis*), precloacal pores in males 4–5 (vs. 7–11 in *bugiamapensis*), SDLF4 18–20 (vs. 15–17 in *bugiamapensis*), SDLT4 20–23 (vs. 17–20 in *bugiamapensis*), CrusL/SVL in adult specimens 0.172–0.200 (vs. 0.144–0.157 in *bugiamapensis*), large nuchal loop bordered anteriorly and posteriorly by broad yellow bands (vs. narrow nuchal loop bordered by distinct narrow white lines in *bugiamapensis*), dark brown body bands 3–4 (vs. seven highly irregular dark blotches with light margins in *bugiamapensis*), and top of head with yellowish spots (vs. distinct dark brown spots in *bugiamapensis*).

*Cyrtodactylusphnomchiensis* sp. nov. differs from *C.caovansungi* Orlov, Nguyen, Nazarov, Ananjeva & Nguyen, 2007 by having SVL 76.1–80.7 mm (vs. 90.4–94.0 mm in *caovansungi*), ventral scales 45–54 (vs. 38–44 in *caovansungi*), femoral pores absent (vs. 6 in *caovansungi*), precloacal pores in males 4–5 (vs. 9 in *caovansungi*), SDLF4 18–20 (vs. 22 in *caovansungi*), and enlarged subcaudals absent (vs. present in *caovansungi*).

*Cyrtodactylusphnomchiensis* sp. nov. differs from *C.cattienensis* Geissler, Nazarov, Orlov, Böhme, Phung, Nguyen & Ziegler, 2009 by having SVL 76.1–80.7 mm (vs. 69.0 mm maximum in *cattienensis*), ventral scales 45–54 (vs. 28–42 in *cattienensis*), SDLF4 18–20 (vs. 12–16 in *cattienensis*), and SDLT4 20–23 (vs. 14–19 in *cattienensis*).

*Cyrtodactylusphnomchiensis* sp. nov. differs from *C.cucdongensis* Schneider, Phung, Le, Nguyen & Ziegler, 2014 by having SVL 76.1–80.7 mm (vs. 55.8–65.9 mm in *cucdongensis*), ventral scales 45–54 (vs. 35–44 in *cucdongensis*), SDLF4 18–20 (vs. 13–18 in *cucdongensis*), and SDLT4 20–23 (vs. 15–20 in *cucdongensis*).

*Cyrtodactylusphnomchiensis* sp. nov. differs from *C.cryptus* Heidrich, Rösler, Vu, Böhme & Ziegler, 2007 by having precloacal pores in males 4–5 (vs. 9–11 in *cryptus*), and precloacal pits in females 1–7 (vs. absent in *cryptus*).

*Cyrtodactylusphnomchiensis* sp. nov. differs from *C.dati* Ngo, 2013 by having SVL 76.1–80.7 mm (vs. 70.1 mm maximum in *dati*), regenerated TaL 56.9–79.1 mm vs. (vs. 50.3 mm maximum, non-regenerated TaL in *dati*), femoral pores in both sexes absent (vs. present in *dati*), nuchal loop continuous (vs. broken in *dati*), and dark brown body bands (vs. irregular dark brown blotches on body in *dati*).

*Cyrtodactylusphnomchiensis* sp. nov. differs from *C.gialaiensis* Luu, Dung, Nguyen, Le & Ziegler, 2017 by having SVL 76.1–80.7 mm (vs. 62.8 mm maximum in *gialaiensis*), precloacal pores in males 4–5 (vs. 9–10 in *gialaiensis*), SDLF4 18–20 (vs. 14–15 in *gialaiensis*), and SDLT4 20–23 (vs. 15–17 in *gialaiensis*).

*Cyrtodactylusphnomchiensis* sp. nov. differs from *C.huynhi* Ngo & Bauer, 2008 by having SDLF4 18–20 (vs. 14–17 in *huynhi*), AGL/SVL 0.451–0.481 (vs. 0.370–0.428 in *huynhi*), ventral scales 45–54 (vs. 43–46 in *huynhi*), precloacal pores in males 4–5 (vs. 7–9 in *huynhi*), dark brown body bands 3–4 (vs. 5–6 in *huynhi*); femoral pores in both sexes absent (vs. 3–8 in *huynhi*), and nuchal loop bordered anteriorly and posteriorly by broad yellow bands (vs. narrow cream margin in *huynhi*).

*Cyrtodactylusphnomchiensis* sp. nov. differs from *C.irregularis* by lacking enlarged triangular tubercles at base of tail (vs. present in *irregularis*), CrusL/SVL 0.172–0.200 (vs. 0.138–0.156 in *irregularis*), LDRT 18–20 (vs. 22–24 in *irregularis*), paravertebral tubercles 31–36 (vs. 38–48 in *irregularis*); ventral scales 45–54 (vs. 38–45 in *irregularis*), SDLF4 18–20 (vs. 15–16 in *irregularis*), SDLT4 20–23 (vs. 18–20 in *irregularis*), dark brown body bands 3–4 (vs. 5–7, mostly as irregular blotches in *irregularis*), and yellowish spots on top of head (vs. distinct dark brown spots in *irregularis*).

*Cyrtodactylusphnomchiensis* sp. nov. differs from *C.kingsadai* Ziegler, Phung, Le & Nguyen, 2013 by having SVL 76.1–80.7 mm (vs. 83.0–94.0 mm in *kingsadai*), enlarged femoral scales 0–8 (vs. 9–12 in *kingsadai*), precloacal pore in males 4–5 (vs. 7–9 in *kingsadai*), and subcaudal scales not enlarged (vs. enlarged in *kingsadai*).

*Cyrtodactylusphnomchiensis* sp. nov. differs from *C.phuocbinhensis* Nguyen, Le, Tran, Orlov, Lathrop, MacCulloch, Le, Jin, Nguyen, Nguyen, Hoang, Che, Murphy & Zhang, 2013 by having SVL 76.1–80.7 mm (vs. 46.0–60.4 in *phuocbinhensis*); precloacal pits in females 1–7 (vs. absent in *phuocbinhensis*), top of head with yellow spots (vs. dark brown spots in *phuocbinhensis*), and dark brown body bands (vs. two dark brown longitudinal stripes or blotches in *phuocbinhensis*).

*Cyrtodactylusphnomchiensis* sp. nov. differs from *C.pseudoquadrivirgatus* Rösler, Vu, Nguyen, Ngo & Ziegler, 2008 by having yellow spots on top of head (vs. dark blotches on top of head in *pseudoquadrivirgatus*) and dark brown body bands (vs. highly irregular body blotches in *pseudoquadrivirgatus*).

*Cyrtodactylusphnomchiensis* sp. nov. differs from *C.sangi* Pauwels, Nazarov, Bobrov & Poyarkov, 2018 by having SVL 76.1–80.7 mm (vs. 56.3 mm maximum in *sangi*), paravertebral tubercles 31–36 (vs. 27–29 in *sangi*), ventral scales 45–54 (vs. 37 in *sangi*), precloacal pores in males 4–5 (vs. 7 in *sangi*), and first dark brown body band complete, second, third, and fourth more irregular (vs. highly irregular bands in *sangi*).

*Cyrtodactylusphnomchiensis* sp. nov. differs from *C.takouensis* Ngo & Bauer, 2008 by having LDRT 18–20 (vs. 9–10 smooth tubercles in *takouensis*); ventral scales 45–54 (vs. 39–40 in *takouensis*), SDLF4 18–20 (vs. 16–17 in *takouensis*), SDLT4 20–23 (vs. 18–20 in *takouensis*), and dark brown canthal stripe absent (vs. present in *takouensis*).

*Cyrtodactylusphnomchiensis* sp. nov. differs from *C.taynguyenensis* Nguyen, Le, Tran, Orlov, Lathrop, MacCulloch, Le, Jin, Nguyen, Nguyen, Hoang, Che, Murphy & Zhang, 2013 by having supralabials 11–13 (vs. 8–9 in *taynguyenensis*), precloacal pits in females present (vs. absent in *taynguyenensis*), SDLF 18–20 (vs. 13–18 in *taynguyenensis*), top of head with yellow spots (vs. dark brown blotches in *taynguyenensis*), and dark brown body bands (vs. black irregular blotches margined by light brown in *taynguyenensis*).

*Cyrtodactylusphnomchiensis* sp. nov. differs from *C.yangbayensis* Ngo & Chan, 2010 by having SDLT4 20–23 (vs. 15–17 in *yangbayensis*) and lacking enlarged subcaudal scales (vs. present in *yangbayensis*).

*Cyrtodactylusphnomchiensis* sp. nov. is most closely related in mitochondrial DNA to *C.ziegleri* Nazarov, Orlov, Nguyen & Ho, 2008 (Fig. 2), but differs in morphology from *C.ziegleri* by having SVL 76.1–80.7 mm (vs. 84.6–93.0 mm in *ziegleri*), paravertebral tubercles 31–36 (vs. 38–46 in *ziegleri*), ventral scales 45–54 (vs. 33–45 in *ziegleri*), HeadL/SVL 0.287–0.305 (vs. 0.263–0.284 in *ziegleri*), ESD/SVL 0.116–0.123 (vs. 0.103–0.113 in *ziegleri*), CrusL/SVL 0.172–0.200 (vs. 0.140–0.168 in *ziegleri*), AG/SVL 0.451–0.481 (vs. 0.390–0.444 in *ziegleri*), eyeD/SVL 0.059–0.067 (vs. 0.053–0.057 in *ziegleri*), top of head with yellow spots (vs. dark brown spots in *ziegleri*), large dark brown nuchal loop (vs. narrow in *ziegleri*), distinct, broad yellow band on anterior margin of dark brown nuchal loop (vs. absent in *ziegleri*), and dark brown body bands bordered by yellowish white or light brown bands about half the width of dark brown bands (vs. light yellow or light brown body bands about same width as dark brown body bands in *ziegleri*).

## Discussion

Mitochondrial DNA serves as a useful but imperfect tool for delimiting species within the *C.irregularis* complex (Nguyen et al. 2017; Pauwels et al. 2018). Two divergent mitochondrial lineages occur within *C.ziegleri*, with both lineages found at its type locality of Chu Yang Sin National Park, Dak Lak Province, and one lineage at Nam Nung Nature Reserve, Dak Nong Province, central Vietnam (Figs 1, 2; Nguyen et al. 2013, 2017; Ziegler et al. 2013; Schneider et al. 2014; Pauwels et al. 2018). Thus, from a matrilineal perspective, some individuals of *C.ziegleri* at Chu Yang Sin are more closely related to those at Nam Nung than to other individuals at Chu Yang Sin. Likewise, two divergent mitochondrial lineages occur within *C.cattienensis* (Fig. 2; Nazarov et al. 2012; Nguyen et al. 2013, 2014, 2017; Schneider et al. 2014; Pauwels et al. 2018), and both lineages of *C.cattienensis* can be found in sympatry at Ta Kou Mountain, Binh Thuan Province, southern Vietnam (Nguyen et al. 2017). The mitochondrial divergences within *C.ziegleri* and *C.cattienensis* are uncorroborated by divergences in morphology (or in one or two nuclear markers; Nguyen et al. 2017; Pauwels et al. 2018), and therefore these each continue to each be treated as single species that harbor considerable intraspecific mitochondrial DNA variation (Nguyen et al. 2017; Pauwels et al. 2018). The processes that resulted in the formation of these divergent mitochondrial lineages within *C.ziegleri* and *C.cattienensis* are unknown, but might be a consequence of a period of past separation of populations by geological or climatic events, the subsequent accumulation of mutations in the mitochondrial genome during isolation, and eventually recontact of these separated populations that today exhibit homogeneous morphology but persistent, divergent mitochondrial genomes (i.e., ancestral polymorphism). Similarly, Nguyen et al. (2017) preferred an explanation of allopatric divergence and subsequent migration into sympatry, rather than sympatric divergence, to explain the co-occurrence of the two mitochondrial lineages of *C.cattienensis* at Ta Kou Mountain. Importantly for this study, the divergent and unique mitochondrial lineage of *C.phnomchiensis* sp. nov. is corroborated by a divergence in morphology from *C.ziegleri* and all other members of the *C.irregularis* complex, and so we posit that *C.phnomchiensis* sp. nov. should be recognized as a distinct species.

Unfortunately, our phylogenetic analysis of the COI gene does not resolve the relationships among *C.phnomchiensis* sp. nov. and the two subclades of *C.ziegleri* (subclades Z1 and Z2; Fig. 2). This means that the resulting polytomy renders *C.ziegleri* as non-monophyletic in our analysis (Fig. 2). This polytomy could be a consequence of a near-simultaneous divergence among these three lineages (i.e., short internodes), but is more likely to be a result of insufficient molecular data (Brennan et al. 2017). Unfortunately, most members of the *C.irregularis* complex have been represented by mitochondrial DNA in previous studies only with <700 bp of the COI gene (Brennan et al. 2017), and hence we were limited in our analyses by available comparative sequence data. Additional sequence data for members of this complex are needed. Specifically, additional mitochondrial data may resolve the polytomy found here among the two subclades of *C.ziegleri* and *C.phnomchiensis* sp. nov., and additional nuclear data can be used to test species boundaries that have been hypothesized from morphological and mitochondrial data (one or two nuclear markers have provided some, but limited, phylogenetic utility; Nguyen et al. 2017; Pauwels et al. 2018).

Phnom Chi consists of an isolated small rocky mountain (peak of 652 m elevation) and a few associated smaller hills, altogether encompassing an area of approximately 4,464 ha within the Prey Lang Wildlife Sanctuary in Kampong Thom and Kratie provinces, Cambodia. The base and lower elevations of Phnom Chi have dry and mixed deciduous forest, whereas upper elevations have large dipterocarp-dominated evergreen and semi-evergreen forest. The current habitat remains in relatively good condition, but this long-overlooked site needs urgent conservation attention. Local communities utilize Phnom Chi for resource extraction, notably the tapping of liquid resin from large dipterocarp trees on the mountain, and small-scale, illegal gold extraction around the base, in addition to forest burning during the dry season (possibly by resin tappers). A small pagoda at the base of the mountain and the scenic beauty of the area (Fig. 7) attracts local and domestic tourists, and will likely attract international tourists in the near future. A second species of lizard, the scincid *Sphenomorphuspreylangensis* Grismer, Wood, Quah, Anuar, Poyarkov, Neang, Orlov, Thammachoti & Hun, 2019, was also recently described from Phnom Chi. Phnom Chi is the only feature with any significant topographic relief in Prey Lang Wildlife Sanctuary, other than some isolated limestone karst blocks in the northern section that have not yielded *Cyrtodactylus* during field surveys (TN, unpublished data). As such, *C.phnomchiensis* sp. nov. may be endemic to the immediate vicinity of Phnom Chi, and together with *S.preylangensis*, underscores the importance of the area for biodiversity conservation. Due to having a small area of occupancy, being relatively uncommon, and experiencing ongoing conservation threats, an assessment of *C.phnomchiensis* sp. nov. by the IUCN Red List of Threatened Species (IUCN 2020) is urgently warranted.

Species diversity of *Cyrtodactylus* in Cambodia is likely to be significantly underestimated. Recently, five species were described within the *C.intermedius* complex from the Cardamom Mountains of southwestern Cambodia (Murdoch et al. 2019), and an additional undescribed species in this complex has been reported from northern Cambodia near the Thai border (Geissler et al. 2019). The species diversity of the *C.irregularis* complex in Cambodia is even less known. Stuart et al. (2006) and Nazarov et al. (2008, 2012) referred to unstudied specimens in the *C.irregularis* complex from Mondolkiri and Ratanakiri Provinces in hilly eastern Cambodia, and those from Ratanakiri Province were later referred by Stuart et al. (2010) to *C.pseudoquadrivirgatus*. The rapid rate of taxonomic partitioning within the *C.irregularis* complex during the past decade, underscored by the realization that many of these newly-recognized species have very narrow geographic ranges, suggests that re-evaluation of the species identities of the Mondolkiri and Ratanakiri specimens is needed.

## Supplementary Material

XML Treatment for Cyrtodactylus phnomchiensis

## References

[B1] Brennan IG Bauer AM Ngo TV Wang YY Wang WZ Zhang YP Murphy RW (2017) Barcoding utility in a mega-diverse, cross-continental genus: keeping pace with *Cyrtodactylus* geckos. Scientific Reports 7: 5592. 10.1038/s41598-017-05261-9 PMC5514027 28717207

[B2] David P Teyníe A Ohler A (2004) A new species of *Cyrtodactylus* Gray 1827 (Reptilia: Squamata: Gekkonidae) from southern Laos. The Raffles Bulletin of Zoology 52: 621–627.

[B3] Geissler P Nazarov R Orlov NL Böhme W Phung TM Nguyen TQ Ziegler T (2009) A new species of the *Cyrtodactylusirregularis* complex (Squamata: Gekkonidae) from southern Vietnam. Zootaxa 2161: 20–32. 10.11646/zootaxa.2161.1.2

[B4] Geissler P Hartmann T Ihlow F Neang T Seng R Wagner P Böhme W (2019) Herpetofauna of the Phnom Kulen National Park, northern Cambodia – An annotated checklist. Cambodian Journal of Natural History 2019(1): 40–63.

[B5] Grismer LL Wood Jr PL Thura MK Zin T Quah ESH Murdoch ML Grismer MS Aung Lin Kyaw H Lwin N (2017) Twelve new species of *Cyrtodactylus* Gray (Squamata: Gekkonidae) from isolated limestone habitats in east-central and southern Myanmar demonstrate high localized diversity and unprecedented microendemism. Zoological Journal of the Linnean Society 182(4): 862–959. 10.1093/zoolinnean/zlx057

[B6] Grismer LL Wood Jr PL Quah ESH Anuar S Poyarkov NA Neang T Orlov NL Thammachoti P Hun S (2019) Integrative taxonomy of the Asian skinks *Sphenomorphusstellatus* (Boulenger, 1900) and *S.praesignis* (Boulenger, 1900) with the resurrection of *S.annamiticus* (Boettger, 1901) and the description of a new species from Cambodia. Zootaxa 4683(3): 381–411. 10.11646/zootaxa.4683.3.4 31715918

[B7] Hayes B Eang HK Neang T Furey N Chhin S Holden J Hun S Phen S La P Simpson V (2015) Biodiversity assessment of Prey Lang: Kratie, Kampong Thom, Stung Treng and Preah Vihear Provinces. Conservation International, Winrock International, USAID, Phnom Penh, Cambodia, 124 pp.

[B8] Heidrich A Rösler H Vu TN Böhme W Ziegler T (2007) Another new *Cyrtodactylus* (Squamata: Gekkonidae) from Phong Nha-Ke Bang National Park, central Truong Son, Vietnam. Zootaxa 1445(1): 35–48. 10.11646/zootaxa.1445.1.3

[B9] IUCN (2020) The IUCN Red List of Threatened Species. Version 2019-3. https://www.iucnredlist.org

[B10] Ivanova NV Dewaard JR Hebert PDN (2006) An inexpensive, automation-friendly protocol for recovering high-quality DNA. Molecular Ecology Notes 6: 998–1002. 10.1111/j.1471-8286.2006.01428.x

[B11] Lanfear R Frandsen PB Wright AM Senfeld T Calcott B (2017) PartitionFinder 2: new methods for selecting partitioned models of evolution for molecular and morphological phylogenetic analyses. Molecular Biology and Evolution 34(3): 772–773. 10.1093/molbev/msw260 28013191

[B12] Luu VQ Bonkowski M Nguyen TQ Le MD Schneider N Ngo HT Ziegler T (2016) Evolution in karst massifs: cryptic diversity among bent-toed geckos along the Truong Son Range with descriptions of three new species and one new country record from Laos. Zootaxa 4107(2): 101–140. 10.11646/zootaxa.4107.2.1 27394811

[B13] Luu VQ Dung TV Nguyen TQ Le MD Ziegler T (2017) A new species of the *Cyrtodactylusirregularis* complex (Squamata: Gekkonidae) from Gia Lai Province, Central Highlands of Vietnam. Zootaxa 4362(3): 385–404. 10.11646/zootaxa.4362.3.4 29245436

[B14] Miller MA Pfeiffer W Schwartz T (2010) Creating the CIPRES Science Gateway for inference of large phylogenetic trees. Proceedings of the Gateway Computing Environments Workshop (GCE): 1–8. 10.1109/GCE.2010.5676129

[B15] Murdoch ML Grismer LL Wood Jr PL Neang T Poyarkov NA Ngo TV Nazarov RA Aowphol A Pauwels OSG Nguyen HN Grismer JL (2019) Six new species of the *Cyrtodactylusintermedius* complex (Squamata: Gekkonidae) from the Cardamom Mountains and associated highlands of Southeast Asia. Zootaxa 4554(1): 1–62. 10.11646/zootaxa.4554.1.1 30790979

[B16] Nazarov RA Orlov NL Nguyen SN Ho CT (2008) Taxonomy of naked-toes geckos *Cyrtodactylusirregularis* complex of South Vietnam and description of a new species from Chu Yang Sin Natural Park (Krong Bong District, Dac Lac Province, Vietnam). Russian Journal of Herpetology 15(2): 141–156.

[B17] Nazarov R Poyarkov NA Orlov NL Phung TM Nguyen TT Hoang DM Ziegler T (2012) Two new cryptic species of the *Cyrtodactylusirregularis* complex (Squamata: Gekkonidae) from southern Vietnam. Zootaxa 3302(1): 1–24. 10.11646/zootaxa.3302.1.1

[B18] Ngo TV (2013) *Cyrtodactylusdati*, a new forest dwelling Bent-toed Gecko (Squamata: Gekkonidae) from southern Vietnam. Zootaxa 3616(2): 151–164. 10.11646/zootaxa.3616.2.4 24758800

[B19] Ngo TV Bauer AM (2008) Descriptions of two new species of *Cyrtodactylus* Gray 1827 (Squamata: Gekkonidae) endemic to southern Vietnam. Zootaxa 1715(1): 27–42. 10.11646/zootaxa.1715.1.2

[B20] Ngo TV Chan OK (2010) A new species of *Cyrtodactylus* Gray, 1826 (Squamata: Gekkonidae) from Khanh Hoa Province, southern Vietnam. Zootaxa 2504(1): 47–60. 10.11646/zootaxa.2504.1.4

[B21] Nguyen SN Le TT Tran TAD Orlov NL Lathrop A MacCulloch RD Le TT Jin J Nguyen LT Nguyen TT Hoang DD Che J Murphy RW Zhang Y (2013) Phylogeny of the *Cyrtodactylusirregularis* species complex (Squamata: Gekkonidae) from Vietnam with the description of two new species. Zootaxa 3737(4): 399–414. 10.11646/zootaxa.3737.4.4 25112761

[B22] Nguyen SN Yang J Le TT Nguyen LT Orlov NL Hoang CV Nguyen TQ Jin J Rao D Hoang TN Che J Murphy RW Zhang YP (2014) DNA barcoding of Vietnamese bent-toed geckos (Squamata: Gekkonidae: *Cyrtodactylus*) and the descriptions of a new species. Zootaxa 3784(1): 48–66. 10.11646/zootaxa.3784.1.2 24872031

[B23] Nguyen SN Zhou WW Le TT Tran TA Jin J Vo BD Nguyen LT Nguyen TT Nguyen TQ Hoang DD Orlov NL Che J Murphy RW Zhang YP (2017) Cytonuclear discordance, cryptic diversity, complex histories, and conservation needs in Vietnamese bent-toed geckos of the *Cyrtodactylusirregularis* species complex. Russian Journal of Herpetology 24(2): 133–154. 10.30906/1026-2296-2019-24-2-133-154

[B24] Orlov NL Nguyen TQ Nazarov RA Ananjeva NB Nguyen SN (2007) A new species of the genus *Cyrtodactylus* Gray, 1827 and redescription of *Cyrtodactylusparadoxus* (Darevsky et Szczerbak, 1997) [Squamata: Sauria: Gekkonidae] from South Vietnam. Russian Journal of Herpetology 14(2): 145–152.

[B25] Pauwels OSG Nazarov RA Bobrov VV Poyarkov NA (2018) Taxonomic status of two populations of Bent-toed Geckos of the *Cyrtodactylusirregularis* complex (Squamata: Gekkonidae) with description of a new species from Nui Chua National Park, southern Vietnam. Zootaxa 4403(2): 307–335. 10.11646/zootaxa.4403.2.5 29690235

[B26] Rambaut A Drummond AJ Xie D Baele G Suchard MA (2018) Posterior summarization in Bayesian phylogenetics using Tracer 1.7. Systematic Biology 67: 901–904. 10.1093/sysbio/syy032 29718447 PMC6101584

[B27] Ronquist F Teslenko M Van der Mark P Ayres DL Darling A Höhna S Larget B Liu L Suchard MA Huelsenbeck JP (2012) MrBayes 3.2: efficient Bayesian phylogenetic inference and model choice across a large model space. Systematic Biology 61: 1–4. 10.1093/sysbio/sys029 22357727 PMC3329765

[B28] Rösler H Vu TN Nguyen TQ Ngo TV Ziegler T (2008) A new *Cyrtodactylus* (Squamata: Gekkonidae) from central Vietnam. Hamadryad 33(1): 48–63.

[B29] Schneider N Phung TM Le MD Nguyen TQ Ziegler T (2014) A new *Cyrtodactylus* (Squamata: Gekkonidae) from Khanh Hoa Province, southern Vietnam. Zootaxa 3785(4): 518–532. 10.11646/zootaxa.3785.4.2 24872243

[B30] Smith MA (1921) New or little-known reptiles and batrachians from southern Annam (Indo-China). Proceedings of the Zoological Society of London 1921: 423–440. 10.1111/j.1096-3642.1921.tb03271.x

[B31] Stuart BL Rowley JJL Neang T Emmett DA Som S (2010) Significant new records of amphibians and reptiles from Virachey National Park, northeastern Cambodia. Cambodian Journal of Natural History 2010(1): 38–47.

[B32] Stuart BL Sok K Neang T (2006) A collection of amphibians and reptiles from hilly eastern Cambodia. The Raffles Bulletin of Zoology 54(1): 129–155.

[B33] Swofford DL (2003) PAUP*: Phylogenetic Analysis Using Parsimony *(and Other Methods). Sinauer Associates, Sunderland, Massachusetts, USA.

[B34] Teyníe A David P (2010) Voyages naturalists au Laos. Les reptiles. Revoir Editions, Chamalieres, France, 315 pp.

[B35] Uetz P (2020) The Reptile Database. http://www.reptile-database.org [Last accessed 13 February 2020]

[B36] Ziegler T Phung TM Le MD Nguyen TQ (2013) A new *Cyrtodactylus* (Squamata: Gekkonidae) from Phu Yen Province, southern Vietnam. Zootaxa 3686(4): 432–446. 10.11646/zootaxa.3686.4.2 26473231

